# On the Use of Warm and Hot Water in Surgery

**Published:** 1875-12

**Authors:** Frederick E. Hyde

**Affiliations:** New York


BUFFALO

### Medical and Surgical Journal.

VOL XV. DECEMBER, 1875. No. 5.

### Original Communications.

———:o:———

ART. I.—*Warm and Hot Water in Surgery. A Short Historical Sketch, with the Present Most Approved Methods of Application. With Cases.* By FREDERICK E. HYDE, M. D., New York.

In 1850, Alphonse Auguste Amussat wrote a thesis upon the "Use of Water in Surgery," (translated by Dr. Frank H. Hamilton, and published in the BUFFALO MEDICAL JOURNAL in 1851), giving an historical sketch of its use from the time of Hippocrates, about 400 B. C., down to his own time, wherein are shown the different periods of its rise and fall in professional favor.

In preparing the following short sketch, I have availed myself largely of Dr. Hamilton's translation above referred to, with a few quotations from Dr. Adam's translation of Hippocrates.

From Hippocrates we learn that in cases of ecchymosis, contusions, stretching and rupture of muscular fibres, in luxations, sprains, diastasis, fractures extending into articulations, etc., etc., after the application of suitable bandages, copious affusions of water are to be prescribed. In luxations of the astragaloss, calcaneum, and in all articular lesions, he recommends warm affusions.

In his Aphorisms, section V.; 22, referring to heat as applied in the form of hot water, he says: "Heat is suppurative, but not in all kinds of sores; but when it is, it furnishes the greatest test of their being free from danger. It softens the skin, makes it thin, removes pain, soothes rigors, convulsions and tetanus. It is particularly efficacious in fractures of the bones, especially of those which have been exposed, and most especially of wounds of the head and in mortifications; in herpes exedens of the arms, of the privy parts, the womb, the bladder, in all these cases, heat is agreeable and brings matters to a crisis, but cold is prejudicial and does mischief."

In the treatise "On the Use of Liquids," considered to be one of the writings of Hoppocrates, we have the same points in reference to the hot water, stated in almost the same words.

In his surgery, he says: "As to the temperature and quantity of the water used, its heat should be just such as the hand can bear, and it ought to be known that a large quantity is best for producing relaxation and attenuation, whereas a moderate quantity is best for incarnating and softening. The limit to the affusion is, to stop when the parts become swelled up, and before the swelling subsides, for the parts swell up at first and fall afterwards."

Speaking of fractures of the forearm, he says: "When you remove the bandages you must pour hot water on the arm and bind it up again." Also in dislocation of the ankle, "At each time that the bandages are taken off, much hot water is to be used, for in all injuries at joints the affusion of hot water in large quantities is to be had recourse to."*

Cornelius Celsus, who lived at the beginning of the Christian Era, and who extolled more than any one else the use of water in internal diseases, and in its external use follows closely the teachings of Hippocrates, says: "A sponge immersed in cold water alone answers in slight cases; but whatever be the liquid with which it is charged it allays pain so long as it is moist, therefore we must not permit it to become dry. In this way we may heal wounds without having recourse to foreign, scarce, and compound medicaments." Farther on he says: "If adhesion has commenced

*F. Adam's Translation of Hippocrates, Sydenham Publication.

and if there is but slight tumefaction, we must adhere to the first kind of dressing; but if inflammation is active, and there is no hope of agglutination we ought to employ suppuratives. The use of warm water is equally necessary to resolve engorgements, to diminish hardness, and to render suppuration more active. The warmth of the water must be such, that the hand when plunged into the liquid shall experience an agreeable sensation, and it is well to continue this application until the wound appears less swollen and has a more natural temperature."

Celsus also recommends the use of water in hæmorrhages, fractures, diseases of the eye, etc.

Galen in the second century in his "Treatise on the Nature and Properties of Simple Remedies," investigates the action of snow and of cold water on the tissues, using in wounds, however, successively warm water, wine, vinegar, recommending especially affusions of warm oils for those cases in which nerves and tendons were implicated.

In the ninth century, Rhazes advises warm water in fractures, and cold water or rose water cooled by ice for burns.

Later, Marianus Sanctus, in a projected but unpublished commentary on the works of Avicenna, intended to teach "a new method of curing wounds, even those of the gravest character, by means of pure water alone only adding thereto certain words, for all the art of medicine consists in words, herbs and stones."

In the sixteenth century, Ambrose Paré, endeavoring to separate superstitious practices from the use of water, says: "I will not omit to say that some cure wounds with pure water after having pronounced over them certain words, and having applied linen cloths cut in the form of crosses and saturated with water often renewed. I affirm that it is neither the words nor the crosses, but it is the water that cleanses the wound, and by its coldness repels the inflammation and the fluxion which might attack the injured part in consequence of the pain. This healing can be accomplished when the wound is in a fleshy part, and in a body young and of good habits, and where the wound is simple."

In 1732, Lamorier read a memoir upon the use of common water for wounds before the "Royal Society of Sciences of Montpelier,"

in which he gives his experience in the treatment of three soldiers; one had an old ulcer on the outer side of the ankle of the size of the hand, another had received a saber blow on the back of the hand, severing all the extensor tendons and separating the fourth and fifth metacarpal bones; this injury had been followed by severe inflammation and abscesses involving the forearm. A third had received a sword cut across the forearm severing the interosseous artery, much blood was effused and extensive suppuration had occurred. For the treatment of the ulcer a copper vessel was constructed for the purpose of immersing the leg, common warm water being used; in this bath the patient rested the limb an hour each day. In a few days the hard borders softened, the cicatrix advanced daily, and he was completely cured. "Two machines of sheet iron were also constructed in which the two soldiers could comfortably immerse the arm from the hand to the elbow. By bathing their wounds in water, suppuration became much more healthy; they were able to move the fingers with greater ease, the pain and the fever diminished daily, in a word, they were entirely cured."

Lamorier was thus the first, as far as we know, to use the prolonged bath in surgery, and with warm water. Gradually the use of the prolonged bath of simple water was forgotten, and it was not till a century later that a Swiss surgeon brought it into notice again.

Cotemporary with Lamorier's use of warm water, cold water was employed in Germany, but applied with cloths kept constantly moist.

"Water," says Guyot, "is the first, the most powerful, and the most universal emollient, I speak of fresh water in its most simple form * * * * It is necessary, then, that water should have a moderate warmth, nearly the temperature of the sound body, in order to produce an emollient effect."

According to the French surgeon Louis, "Luke-warm water, is of all medicaments the most simple. Yet we derive from it benefits without number; luke-warm water relaxes parts which are overstretched, opens the pores, the particles of water insinuate themselves into the vessels, dilute the fluids, and increase the diameter of the small invisible vessels, they facilitate the flow of humors, and open passages to substances which need be expelled. It is for all these reasons that Paré recommends fomentations of luke-warm water in several places, and especiallly in the thirtieth chapter of the fifteenth book upon 'fractures.'"

The most notable cases of success resulting from the use of water in gunshot wounds, are those reported by Dr. Treille, after the battle of Baylen. He says: "After the battle of Baylen (Andalusia) I remained upon the field the only surgeon to take care of five hundred wounded. Deprived of all medicines, I had all the wounds washed with pure water. I continued my dressings in this way during twenty-one days that we remained upon the battle field, receiving nothing from without but some linen and provisions. As it would be impossible for me alone to have dressed five hundred wounded, I arranged them in three sections and dressed one section each day, and they dressed themselves the two other days. Only seven or eight wounds became gangrenous, and I had but two cases of tetanus.

"When attention is given to the circumstances in which I was placed, it will be apparent what we ought to think of simple water in the treatment of recent wounds. Here were five hundred wounded lying upon the ground from the nineteenth of June to the tenth of July, (1808) under the broiling sun of Andalusia, having nothing whatever for shade but the thin branches of olive trees, deprived of the consolatory hope of ever again seeing their own country, and given up to the mercy of the inhabitants of the Sierra Morena, who were all in arms and highly exasperated.

"In a word, the moral as well as the physical, was but little favorable to the treatment of wounds; I have shown you nevertheless what was my success."

Guthrie recommends cold water compresses in gunshot wounds, but does not consider it an infallible remedy in all cases of inflammation. When it produces disagreeable sensations upon its first application, such as shiverings and stiffness of the part, and when it does not soothe, it is doing harm and warm water should take its place.

Sanson in 1831, says: "With water I have seen cured by first intention contused wounds, accompanied with more or less laceration and stretching of the parts; I have been able to save most persons upon whom I have practised amputations, or other grave operations, from the so-called traumatic fever; indeed, I have been able to cure, without amputation, and even without active inflammation or copious suppuration, many persons having fractured limbs complicated with wounds and projection of the fragments."

A few years previous to 1830, Josse, of Amiens, instituted irrigations as a method of applying water to wounds, that is, allowing the water to flow upon the wound from a vessel placed above it. In 1834, Breschet introduced this method at the Hotel Dieu at Paris, with considerable success.

In his "Operative Surgery," 2d Ed., 1839, Velpeau, who was opposed to the use of irrigations, says: "I think I can predict, after what I have seen, that irrigations will not continue to be used except as an occasional modification of the dressings and in a small number of cases."

In 1841, Charles Mayor published his method of applying baths to all parts of the body, the vessel being shaped to fit the irregularities of the part to be treated. Rubber was applied to the edges of the vessel to prevent the escape of the water. He speaks of the use of warm water only in these baths.

In 1844, Nélaton, speaking of the treatment of contused wounds in his "Elements of Surgical Pathology" thus expresses himself: "Cold water has not always been employed in the same manner in the treatment of wounds: sometimes there is applied upon the wounds one or several compresses dipped in this liquid, and when they begin to become warm they are removed, or dipped again in cold water. This method exposes the parts to alternations of heat and cold, either by a complete omission to renew the cloths, or by their not being renewed sufficiently often; this method has therefor been abandoned, and continued irrigations with cold water have been substituted * * * * * Nevertheless in spite of its advantages, permanent irrigation with cold water cannot be employed as a general method of treatment. All who have employed it regard it as an exceptional method, especially applicable to contused wounds, and more epecially to wounds complicated with fracture, where, as in the case of the upper extremity, the injury is not above the elbow, and in the case of the lower extremity, not above the knee.

MM. Breschet, A. Berard and Pinel Grandchamp have substituted tepid water for cold in irrigation, and the results have been generally satisfactory."

Amussat further states that his father constantly made use of water in the treatment of wounds and complicated fractures, and in fact in most surgical affections and after operations.

He concludes that cold water (50° to 32° F.) applied continuously upon inflamed parts is a powerful anti-phlogistic and sedative, to be used with great caution and discrimination as to the ability of different patients to support it, as, in his own experience it has produced chills, too sudden suppression of the inflammation, and gangrene. It is consequently recommended in the treatment of the young and robust, and to be avoided in the treatment of infants and the aged, for whom water is to be employed at a temperature of 86° to 95° F. Its use is recommended in simple inflammations, erysipelas, burns, ulcers, gangrene, wounds, simple and contused, gunshot wounds, wounds from operations, amputations, etc., hæmorrhages, contusions, affections of joints, hernias, diseases of the eyes, diseases of the genital and urinary organs. This list it will be observed corresponds largely with the conditions for which the use of *warm water* is recommended by Hippocrates.

As to the mode of applying water, Amussat recommends the *water dressings* as a simple means of application, and suitable for superficial injuries, and for those parts that cannot be readily irrigated or immersed. *Irrigations* are more powerful than dressings, but often impossible to apply; when used they are simply superficial in their action.

*Immersion*, however, has a more prompt and decided effect than either of the other methods, and is advised for the immediate treatment of the most severe surgical accidents, as the water penetrates to the deeper tissues, cleansing the wound, and encouraging granulation. The temperature is to be maintained at about

64° to 68° F., the treatment to be terminated with water dressings.

Within the past few years water treatment has again attained some prominence. Billroth, in his "Surgical Pathology,"* prefers *immersion* and *water dressings* to irrigation, as being more simple and convenient. He has used immersion in both the "continued cold-water bath," kept cold by placing ice in it, and the "warm bath," in the treatment of contused wounds of hand, forearm, foot and leg. "In most cases of these injuries the bleeding is so slight, and ceases so soon spontaneously, that the patient can place the extremity under water very soon, if not immediately, after the injury, without the occurrence of hæmorrhage; but the blood clinging to the part should first be washed off, the water itself be perfectly pure and transparent, and, if it becomes clouded by the secretion of the wound, it should be kept clear by frequent renewals." He considers "that there is very little difference in the effect of the water upon the wound with a temperature varying from 54° to 90° or 100° F., although the higher temperature may induce suppuration a little sooner." He therefore "uses that temperature which is most agreeable to the patient." This, I have observed, is generally from 95° to 100° F. Billroth calls attention to one point in the treatment of the hands and feet which I have observed in my practice. In some persons, after the hand has been submerged for three or four days, an intense burning pain is experienced along the lines of depression on the palmer surface, the intermediate space being swollen into dense, hard ridges, making the water application unbearable. This may be remedied, or avoided, by putting a handful of salt in the water. Whatever the true explanation of the pathology of this condition may be, it would seem to be due to great tension made upon the subcutaneous, inelastic fibrous tissue peculiar to these parts by the swelling of the looser tissue into hard ridges, as also the compression of the nerve terminals, which are so numerous on the palmer surface of the hand and fingers. The addition of the salt to the water causes the ridges to disappear and relieves the tension and pain.

Billroth kept the limb immersed from eight to twelve days, after

*Dr. C. E. Hackley's translation. 1870.

which water dressings, moist cloths covered with oiled silk, were applied till the wound healed.

Billroth refers the methodizing of water treatment to B. von Langenbeck.

As a more complete exposition of the methods and results of treatment by submersion employed during the past few years in Germany, I have drawn largely from a small pamphlet "On Injuries of the Hand and Fingers," by Dr. Max Schede, First Assistant to the Surgical Clinic at Halle.

This paper was published in December, 1871, and although now four years old, it contains many instructive facts and suggestions. Dr. Schede did not use water at a high temperature, and therein failed to obtain all the benefits of water in suppurating wounds. He recommends a temperature, at first, of 20° C. (68° F.) to stop parenchymatous hæmorrhage, which, of course, is eminently proper, but incidentally remarks, "the patients are unable to bear it longer than a few hours, when they begin to feel very cold, and urgently ask for warmer water. Afterwards the temperature is to be raised to 25° to 30° C. (77° to 86° F.). This temperature, it will be observed, is not quite that of warm water, 90° F.

As by the general methods of surgical practice, about fifty per cent. of severe injuries of the fingers, such as lacerated wounds, compound fractures, etc., result in the loss of those members, and as the loss or usefulness of the hand or some fingers is of primary importance to those dependent upon manual labor for their support, it becomes the duty of the surgeon to avail himself of every means at his command not only to retain these members, but also to endeavor to restore their function. With the use of submersion in the water-bath, 100° F., I do not doubt but that this loss may be reduced one-half.

Omitting the minor and simpler lesions and taking up the worst cases only, Dr. Schede, in presenting the subject, supposes the case of a strong man who has had the misfortune to get his hands into the wheels of a piece of machinery. The injury is recent, some phalanges, or one or more fingers, are completely crushed so as to be without any vitality; other parts are less injured, but have been subjected to compound fracture; some joints are opened with or without dislocation, and the skin is more or less bruised and broken, hanging in flaps or entirely wanting.

The first inquiries of the surgeon naturally are, what must be removed and what might be preserved? Undoubtedly some parts are without vitality, and gangrene will occur; some of the fingers are already gone, and none of the remainder free from injury. Is it advisable to expose the patient to suppuration of the joints and purulent resorption, in the uncertain hope of preserving the use of a finger and a portion of one or two more? If the surgeon decides to amputate he must make his incisions entirely in healthy tissue, and thus sacrifice some portions capable of preservation. If he operates in the injured parts, he leaves tissue which will, in all probability, soon be a source of infection to the whole body. If the inflammatory period sequent to reaction have already occurred, he must wait for the secondary period, or period of suppuration, to arrive. With the water treatment, however, Schede directs the removal, primarily, of only those parts which are utterly destroyed, leaving the subsequent separation of gangrenous portions entirely to nature. The judicious conduct of this process, especially by permanent immersion, entails no more danger than primary amputation. The danger of pyæmia is not increased by this delay, it having occurred but very seldom, even in hospitals, in the course of this treatment. Billroth has observed pyæmia in Zurich but three times in 277 cases, most of which were treated by the permanent water-bath. In Halle, where the hospital is poorly ventillated and badly built, there were but two cases in forty, both of which were infected from some outside source after the wounds were clean and granulating. In the first case pleurisy was the first symptom, and the patient died. The second case was saved by amputation.

As this case is interesting and instructive, I will give Dr. Schede's entire record of it:

A strong, healthy man, aged thirty-two years, had his left hand injured in some machinery, to such an extent as to necessitate his removal to the hospital, June 26, 1869.

A contused and lacerated wound was found extending from the ulnar side of the wrist across the dorsal surface of the hand to the metacarpo phalangeal joint of the index finger, and thence passing around the palmer surface, across which it extended to the middle of the fifth metacarpal bone. The extensior tendons were partiallly torn; the second, third and fourth metacarpal bones were fractured in several places, and partially dislocated, the soft parts much separated towards the arm. The metacarpo-phalangeal joint of the index finger was opened upon the radial and palmer aspects, while the skin of the palmer surface was entirely separated, forming a large flap.

As we had previously obtained good results in worse cases with the expectant treatment, we resolved to pursue the same course in this case; the broken bones were replaced so far as practicable, the hand placed upon a shingle and put in the permanent bath.

Reaction was at first moderate; temperature rose in the morning to 38.5° C. (101.3° F.); in the evening it averaged from 39° to 39.5°C. (102.2° to 103.1° F.). The wound, especially upon the dorsal surface, was covered with gangrenous masses. The flap on the palmer surface became attached. On the sixth day the index finger was entirely gangrenous, and was removed at the metacarpal joint, near the line of demarcation, but still in the gangrenous portion. On the eighth day also the second and third phalanges were removed from the second finger.

On the tenth day, the wound was entirely clean, granulations appeared, and the hand was removed from the water-bath and dressed with a solution of carbolic acid, (1 to 100.) The temperature rose the same evening to 40.5° C. (104.9° F.). Next morning a chill occurred, lasting half an-hour. No retention of pus, patient looks somewhat yellow, is covered with cold sweat, general state of health very bad, temperature 40.5° C. (104.9° F.). Amputation of arm near the elbow-joint through healthy tissue, formation of two flaps. The wound is closed six hours later, and the patient removed outdoors. Temperature in the evening 37.2° C. (98.9° F.), pulse 60.

Thirteenth day.—Temperature 37.2° C. (98.9 F.), pulse 76 small, no return of sweat. Evening temperature 38.4° C. (101.1° F.), pulse 60.

Fourteenth day.—Temperature, morning, 38.6° C. (101.5° F.); evening, 39.2° C. (102.6° F.).

Fifteenth day.—Temperature, morning, 38.4° C. (101.1° F.); evening, 39.4° C. (102.9° F.)

Nowhere was there union by first intention, as a small margin of the flaps became gangrenous. Temperature was normal on the eighth day after amputation. Three days later, two abscesses formed in the subcutaneous cellular tissue, one over the right sterno-clavicular articulation, the other in the right natis. Both formed rapidly; the first contained laudable, the other bad-smelling pus. They closed soon, and the man recovered soon afterwards."

Inasmuch as the temperature rose as soon as the hand was removed from the water, should it not have been immersed again immediately that the accession of temperature was observed? and I venture the suggestion that if hot water, 100°–105° F., had been employed, the infection would not have occurred.

The rule at Halle is to immediately put the wounded hand in the bath, and then to wait till the wound is clean and every gangrenous portion has come away, or at least till the line of demarcation is formed, and the temperature of the patient nearly normal, before removing it. However, gangrenous tissues, loose and devitalized phalanges, or entire fingers, are removed with the scissors, in order to keep the atmosphere of the ward as pure as possible. In some cases it is well at this time to perform any operation that may be necessary to hasten recovery. In others, the exfoliation of necrosed bone is awaited, or the effect of cicatricial contraction is observed, so as to be able to leave but little interference at a later period; although, by this treatment, the postponement of an operation has no danger,

In amputations following the above treatment, Dr. Schede observes that the flaps must be made rather large, on account of the distention and infiltration of the integuments. He has found that the results of these secondary operations are mostly very favorable, the danger only nominal. Union by the first intention generally takes place, if the flaps are carefully united, due to the infiltration of the tissues with plastic material.

The water treatment is of especial advantage in injuries of the tendinous sheaths, which are naturally of frequent occurrence in injuries of the hand, and are of importance on account of the danger to the patient, and the fact that in healing there is a liability that the sheaths will unite with their tendons, thus destroying the usefulness of the fingers.

The following cases in point, of recent injury treated by submersion, are given in Dr. Schede's paper:

Case I.—A boy, 14 years of age, healthy, placed a loaded pistol in his pocket, the muzzle pointing upwards; in pulling it out the pistol was discharged, the ball passing through his left hand. The patient was sent immediately to the hospital (1st of August).

The middle and ring fingers, with their metacarpal bones, were shattered to pieces, and completely severed from their carpo-metacarpal articulation, both of which were already open. The tendon of the extensor indicis was exposed over the metacarpal bone of the index finger, and was lacerated, but it was possible to cover it with a piece of skin kept in position by a few sutures. The joint between the last metacarpal bone and the unciform was opened on the radial side; the sheath of the flexor minimi digiti was also opened. The thumb alone was uninjured.

Hæmorrhage was stopped carefully with ligatures, lint applied, the hand fastened to a splint and placed in a water-bath, to which some carbolic acid was added. Reaction was at first quite severe; the temperature rose in the evening to 40° C. (104° F.). After seven days the wound was clean. The hand was then taken out of the bath and dressed with a solution of carbolic acid (1 to 100). No excessive inflamation or extensive suppuration took place. Notwithstanding the opening of three carpo-metacarpal joints, none of the neighboring joints were inflamed; no suppuration of the sheaths of the tendons occurrred. The carpo-metacarpal joint of the little finger closed and healed with perfect mobility. Subsequently two small subcutaneous abscesses formed, one on the palmer surface of the forearm close to the wrist joint, the other on the dorsal surface three inches higher up. They, however, had nothing to do with suppuration of sheaths of tendons, and healed soon after they were opened. The patient was discharged September 10th, with a superficial granulating wound, his fingers being still kept on the shingle splint in order to prevent lateral deviation of the index and little fingers. Both fingers and thumb are capable of active motion.

Case II.—A young man, 19 years of age, injured his hand severely in machinery July 7th, 1869, and was brought immediately to the clinic. A smooth incised wound was found, beginning one inch from the right wrist-joint and passing arch-shaped over the dorsum of the forearm, entering and opening the wrist-joint. All along the wound *all the extensor tendons* are cut through. One artery had to be tied. The hand was flexed moderately, placed in a splint and put in the permanent water-bath. This treatment was continued for two weeks. The wound healed by first intention, except the point through which the ligature passed. On the dorsum of the hand an abscess formed, the only one in this case, otherwise there was no extensive suppuration. August 20th, the wound is completely cicatrized. In the beginning of September, cautious, passive motions of the hand were made for the first time, and to a moderate extent without pain. The fingers were quite stiff, having been kept immovable for so long a time, but they soon gained their former mobility. Active motion was soon commenced with the wrist-joint, but unfortunately the patient had to be discharged too soon, and not being careful enough, anchylosis of the joint occurred which might have been avoided.

The next case concerns more the arm than the hand, but will supply additional testimony in favor of submersion.

Case III.—A healthy man, aged thirty-two years, received an injury of his left hand and forearm from a cogwheel, the impressions from the teeth of the wheel being visible at regular intervals along the entire arm. They are mostly superficial, except upon the anterior surface of the forearm, where they are quite deep, and along the middle portion perforations having been made through which muscles and tendons protrude; between the perforations there are spaces of intact skin.

No bone being injured, it was a question whether to amputate or attempt conservative treatment. Probably the larger portion of the flexors would be lost. Possibly a movable thumb might be preserved to the patient subject to the action of its short muscles.

It was finally resolved to try and save the arm. It was placed on a splint and put in the water-bath.

During the next six days great masses of gangrenous tissue came away. The skin mortified to a greater extent than was at first anticipated. The loss of skin is five by three inches. All the flexors, with the exception of the flexores carpi only, have sloughed and are about to come away.

After fourteen days the wound has become perfectly clean. A piece of the median nerve had mortified. An abscess had formed on the upper arm in consequence of the primary contusion, but it soon closed after incision. Otherwise no extensive suppuration occurred. The patient is able to flex and extend the hand; unfortunately, he can move the thumb no more than the fingers. The arm was removed from the water and treated with water-dressings.

Eight days after removal from the bath the patient's temperature was normal, and he left his bed. Cicatrization now advanced rapidly. The tendency of the fingers to flexion is counterbalanced by dorsal splints.

Unfortunately, the patient's family affairs compelled him to leave the hospital too soon. At this time, nine weeks after the injury, the wound was only about one inch and a half in diameter; the fingers were a little movable, probably by the lumbricales muscles; the thumb was immovable and entirely useless.

These cases show what good results may be obtained in very severe injuries by the use of continued submersion.

In some general directions for the use of the water-bath, Dr. Schede observes that it is of use only in recent cases, and that if suppuration is already established, and especially if canals and cavities have formed lodging pus, he says: "It is not only not favorable, but also pernicious. Wounds in this condition placed in the water-bath could neither be kept clean nor free from the secretions. The soft parts and the granulations swell, the openings are diminished in size, and narrow canals closed. Even if free openings are maintained, pus will not flow out freely; as the water coagulates it, it is retained in the wound, and finally decomposes. The granulations become œdematous, assume a pale, smooth appearance, and are unfit for cicatrization."

When Dr. Schede wrote the above, it must be remembered that he was using water at a temperature at the highest, of 30° C. or 86° F. only. Our experience at the present day with hot water is, that just such cases can be treated successfully by submersion; as instance, Dr. Hamilton's "Case XI" in the following pages, in which erysipelatous inflammation and gangrene had occurred before submersion; and the following two cases, which will suffice to demonstrate this point.

The first, which Dr. Hamilton has kindly furnished me, is from his private practice, and is as follows:

Mrs. A. B., aged about 60, had plunged an ice-pick into the interdigital commissure between the ring and middle fingers of one of her hands. Inflammation having been set up, she commenced the application of ice; this she continued assiduously for several days, the inflammation meanwhile progressing steadily.

On about the seventh day, poultices were substituted for the ice. About the ninth day, the case was seen by Dr. Hamilton. The whole hand was found to be inflamed, and suppuration had already commenced. It being, however, late at night, and the operation of incision exposing somewhat to the danger of wounding important arteries, it was determined to defer the opening of the abscess till the following morning; but as she was suffering greatly from a burning or scalding sensation, as she expressed it, she was directed to keep the hand immersed in water at a temperature of 100° F. or above. In the morning, although no matter had escaped, she remarked that her hand was almost free from pain, and that the scalding sensation had entirely ceased the moment she had submerged the hand in the hot water.

Dr. Hamilton opened the abscess, which discharged a considerable amount of pus, and submersion, alternating with hot-water fomentations, were continued.

Two weeks later submersion had been discontinued, and fomentations only were being used; the swelling had gone down and the wound was healing.

The second case occurred in the private practice of Dr. A. Rose, of this city, who has kindly sent me the history of it, as follows:

Fracture of the left malleolus externus; abscess of the leg; necrosis of the fibula with sequestrotomy; sero-fibrinous synovitis of the tibio-tarsal articulation; chronic periostitis of the right clavicle.

Mary Wey, four years of age, fell from a wagon on June 29th, 1875. The physician who first took charge of the case thought he recognized a fracture of the external malleolus, though he was not positive. He applied wooden splints and bandaged the leg; gave internally quinine and calomel. While thus bandaged the child suffered considerably, and the splints were therefore removed July 2d. The suffering continued, and I was called to see the patient in consultation July 3d. Found the foot turned inward, very much swollen and painful; high fever. After correcting the position of the foot, I applied a plaster-of-Paris bandage; cut it open all along the dorsum of the foot and anteriorly along the leg, and suggested that the limb should be kept in a raised position higher than the rest of the body, that ice should be applied at the ankle-joint and quinine freely given. When I left the child felt, or appeared to be, very much relieved. On July 7th I was again called, when I found the child apparently suffering the most excruciating pain. The leg had not been elevated, no ice was applied. I widened the bandage, which was pressing at some points, raised the leg according to the old teaching of Celsus, and the now famous recommendation of Volkman concerning inflammation of the joints, and again had the satisfaction of seeing the child relieved.

July 9th, I called again, and at the request of the physician who had attended the case so far, I took charge of it entirely. Removing all bandages, I found that since my last visit phlegmonous inflammation had developed extensively along the outer side of the leg, and fluctuation could be felt. I made an incision, through which an enormous amount of pus was discharged. I placed the limb in a warm-water bath, and had the satisfaction of finding it promptly relieved from pain. The submersion was continued day and night for four days, until the child began to dislike it, by which time the phlegmonous inflammation had disappeared. It was most interesting to see how the water had separated the gangrenous tissue from the healthy parts, and washed it from the wound, leaving the healthy tissues with a bleached appearance. The wound soon filled up with healthy granulations, a new plaster bandage was applied, and when it was removed at the end of the fifth week after the receipt of the injury, the foot was found to have resumed and to have retained its natural position; in the region of the ankle-joint there was some expansion, but altogether the process of healing seemed to be going on favorably.

After about another fortnight, it was found that at the lower end of the cicatrix of the incised wound there appeared a fistula, and later, at the other extremity, another fistulous opening. By means of a probe introduced I discovered necrosed bone, and as soon as movable, kindly assisted by Dr. Hyde, I removed through the lower fistula a portion of the necrosed fibula five inches in length. The sequestrotomy was performed September 8th. All wounds now closed promptly, but about the 20th of September a little circumscribed swelling appeared over the external malleolus; it was fluctuating, opened spontaneously, and discharged a small quantity of sero-fibrinous matter, the nature of which and the point from which it came made it evident that sero-fibrinous synovitis had developed. Since the removal of the necrosed fibula the patient has suffered but little pain.

For some weeks after I first had charge of the case there was bronchitis present which alarmed me considerably; she had been coughing for nine weeks. I ordered quinine and iron, and stimulated as much as possible. A most peculiar incident in the case was the appearance of a periostitis of the right clavicle, which was noticed shortly after the occurrence of the accident. The periostitis is still persistent, and does not seem to be painful, though for the past few days I have noticed that the swelling has become red, with temperature increased.

Dr. Rose adds in his note to me that, notwithstanding the synovitis at the ankle-joint, the child is walking, bearing her weight upon the sound limb and balancing herself by just touching the toes of the injured foot to the ground. He does not care to keep her in an immovable apparatus indoors, as with a simple roller about the limb she goes out, gets the air, and is improving in general health, to the advantage of the diseased joint.

Dr. Schede insists that the hand shall not hang down in the water, but that the entire forearm must also be submerged, the hand and elbow being at least upon the same level. He finds submersion from six to eight days sufficient, as a rule, for the hand and fingers, at the end of this period the granulating process or exudation of plastic lymph is so far advanced that the limb may be removed from the bath. "It has accomplished its purpose, it has helped to carry the patient over the period of acute reaction, and establish a healthy action in the wound, and its further use would unnecessarily prolong the healing process."

I have observed, however, in the treatment of severe injuries of the lower extremities, that it has generally been found avisable to continue submersion for a longer period than six or eight days, fourteen days being about the average time required, and occasionally longer.

Hueter, of Graefswalde, in his work on "Diseases of the Joints," 1871, speaking of the action of cold and heat, says: "Cold interferes with the process of resorption of inflamed products, and must not be applied if the object is to promote such resorption. If we are treating a joint already inflamed for several months, the process of healing can only be accomplished by the fatty degeneration and resorption of the inflammatory new formation, and of the hydropic synovia, and it is not apparent why the ice should be continued. At most, it will neither retard nor encourage this process, for resorption is brought about by a stimulation of the lymphatic system, which requires a high pressure in the blood-vessels. By this pressure a more active circulation of the nutritive fluid is obtained, and supplied more freely to the lymphatics, and thus, physiologically, warm applicaltions are indicated instead of cold. Cloths moistened in warm water, enclosed with a bandage, if pressure is required, and covered with some impermeable material to keep the temperature, are the most advantageous means to be employed in these inflammations."

Further on, Hueter says: "That neither the warm water-bath nor the ice are sovereign remedies; they both have their place in the reduction of inflammation. When used unintelligently, however, they may both be harmful.

"Warmth is an antiphlogistic indirectly. In some cases, one might think that ice would be selected for its anæsthetic action, where, as the result of experience, warmth is to be preferred. As when we find intense pain, no local or general increase of temperature, and no swelling; and where we must suppose a neuralgia of the synovial membrane; ice is not tolerated, no improvement is obtained, and the pain is actually increaeed.

"Now, what is the explanation of this? It is difficult to answer; but from experience it may be said, that in such chronic inflammations of joints with very little swelling, no increase of temperature and very great painfulness; where the ice acts neither as antiphlogistic nor as anæsthetic, it must be substituted in either case by warmth."

In his "General Surgery," published in 1873, in presenting his view of the action of warmth in connection with hyperplastic inflammation of the deeper tissues, Hueter says: "As soon as they have come to a chronic condition they will give us a double task to perform: 1st, to do away with the danger which is apt to befall the seat of inflammation; and 2d, as soon as suppuration has occurred, to promote the resorption of the hyperplastic formation.

"For resorption of hyperplastic inflammation, ice is of no use. The reduction of temperature causes a contraction of the small arteries, and their reduced capacity will allow but a small quantity of blood to reach the centre of inflammation, supposing, of course, that the cold will reach the arteries of the deeper tissues.

"Now, what is gained by anæmia of the smaller arteries, in the diminution of the extent of inflammation, is lost by the obstruction and retardation of the circulation in the veins, and especially of the lymphatic circulation. The cells of the granulation tissue, and the nutritive fluid which is collected in and between them, having their natural and most important return channels in the lymphatics, will circulate more easily with a high arterial pressure than with a low pressure. The arteries and capillaries being dilated by the application of heat, assisted by the action of the heart, the blood is caused to flow into them in increased quantity, thereby favoring the transportation of the nutritive fluid into the lymphatic vessels; therefore, warmth is to be reckoned among antiphlogistic means, at least in the chronic state of hyperplastic inflammation."

TO BE CONTINUED.

